# Tracking the progressive spread of the SARS-CoV-2 Omicron variant in Italy, December 2021 to January 2022

**DOI:** 10.2807/1560-7917.ES.2022.27.45.2200125

**Published:** 2022-11-10

**Authors:** Paola Stefanelli, Filippo Trentini, Daniele Petrone, Alessia Mammone, Luigina Ambrosio, Mattia Manica, Giorgio Guzzetta, Valeria d'Andrea, Valentina Marziano, Agnese Zardini, Carla Molina Grane’, Marco Ajelli, Angela Di Martino, Flavia Riccardo, Antonino Bella, Monica Sane Schepisi, Francesco Maraglino, Piero Poletti, Anna Teresa Palamara, Silvio Brusaferro, Giovanni Rezza, Patrizio Pezzotti, Stefano Merler, the Genomic SARS–CoV–2 National Surveillance Working Group, the Italian Integrated Surveillance of COVID–19 Study Group, Genomic SARS-CoV-2 National Surveillance Working Group, Alessandra Lo Presti, Stefano Morabito, Gabriele Vaccari, Ilaria Di Bartolo, Arnold Knijn, Luca De Sabato, Liborio Stuppia, Giovanni Savini, Antonio Picerno, Teresa Lopizzo, Domenico Dell’Edera, Pasquale Minchella, Francesca Greco, Giuseppe Viglietto, Maria Teresa Fiorillo, Luigi Atripaldi, Antonio Limone, Davide Cacchiarelli, Pierlanfranco D’Agaro, Danilo Licastro, Stefano Pongolini, Tiziana Lazzarotto, Giada Rossini, Vittorio Sambri, Giorgio Dirani, Silvia Zannoli, Paola Affanni, Maria Eugenia Colucci, Maria Rosaria Capobianchi, Florigio Lista, Anna Anselmo, Patricia Alba, Alice Massacci, Carlo Federico Perno, Maurizio Sanguinetti, Bianca Bruzzone, Giancarlo Icardi, Flavia Lillo, Andrea Orsi, Elena Pariani, Fausto Baldanti, Maria Rita Gismondo, Valeria Micheli, Fabrizio Maggi, Arnaldo Caruso, Ferruccio Ceriotti, Maria Beatrice Boniotti, Ilaria Barbieri, Alice Nava, Erminio Torresani, Fabiana Cro, Enzo Boeri, Marina Noris, Giulia Bassanini, Claudio Farina, Marco Arosio, Sergio Malandrin, Annalisa Cavallero, Patrizia Bagnarelli, Stefano Menzo, Silvio Garofalo, Massimiliano Scutellà, Elisabetta Pagani, Lucia Collini, Valeria Ghisetti, Silvia Brossa, Giuseppe Ru, Elena Bozzetta, Maria Chironna, Antonio Parisi, Salvatore Rubino, Sergio Uzzau, Flavia Angioj, Gabriele Ibba, Caterina Serra, Giovanna Piras, Giuseppe Mameli, Ferdinando Coghe, Francesco Vitale, Fabio Tramuto, Guido Scalia, Concetta Ilenia Palermo, Giuseppe Mancuso, Teresa Pollicino, Francesca Di Gaudio, Stefano Vullo, Stefano Reale, Maria Grazia Cusi, Gian Maria Rossolini, Mauro Pistello, Antonella Mencacci, Barbara Camilloni, Silvano Severini, Massimo Di Benedetto, Calogero Terregino, Alice Fusaro, Mosè Favarato, Laura Squarzon, Isabella Monne, Valeria Biscaro, Italian Integrated Surveillance of COVID-19 Study Group, Martina Del Manso, Matteo Spuri, Chiara Sacco, Massimo Fabiani, Marco Bressi, Alberto Mateo-Urdiales, Maria Fenicia Vescio

**Affiliations:** 1 Istituto Superiore di Sanità, Rome, Italy; 2 Center for Health Emergencies, Bruno Kessler Foundation, Trento, Italy; 3 Dondena Centre for Research on Social Dynamics and Public Policy, Bocconi University, Milan, Italy; 4 Health Prevention Directorate, Ministry of Health, Rome, Italy; 5 Epilab–JRU, FEM–FBK Joint Research Unit, Trento, Italy; 6 University of Trento, Trento, Italy; 7 Laboratory for Computational Epidemiology and Public Health, Department of Epidemiology and Biostatistics, Indiana University School of Public Health, Bloomington, United States; 8 The members of the Genomic SARS–CoV–2 National Surveillance Working Group are listed under Collaborators; 9 The members of the Italian Integrated Surveillance of COVID–19 Study Group are listed under Collaborators

**Keywords:** omicron, doubling time, prevalence, genomic survey, SARS–CoV–2, COVID–19

## Abstract

**Background:**

The SARS-CoV-2 variant of concern Omicron was first detected in Italy in November 2021.

**Aim:**

To comprehensively describe Omicron spread in Italy in the 2 subsequent months and its impact on the overall SARS-CoV-2 circulation at population level.

**Methods:**

We analyse data from four genomic surveys conducted across the country between December 2021 and January 2022. Combining genomic sequencing results with epidemiological records collated by the National Integrated Surveillance System, the Omicron reproductive number and exponential growth rate are estimated, as well as SARS-CoV-2 transmissibility.

**Results:**

Omicron became dominant in Italy less than 1 month after its first detection, representing on 3 January 76.9–80.2% of notified SARS-CoV-2 infections, with a doubling time of 2.7–3.3 days. As of 17 January 2022, Delta variant represented < 6% of cases. During the Omicron expansion in December 2021, the estimated mean net reproduction numbers respectively rose from 1.15 to a maximum of 1.83 for symptomatic cases and from 1.14 to 1.36 for hospitalised cases, while remaining relatively stable, between 0.93 and 1.21, for cases needing intensive care. Despite a reduction in relative proportion, Delta infections increased in absolute terms throughout December contributing to an increase in hospitalisations. A significant reproduction numbers’ decline was found after mid-January, with average estimates dropping below 1 between 10 and 16 January 2022.

**Conclusion:**

Estimates suggest a marked growth advantage of Omicron compared with Delta variant, but lower disease severity at population level possibly due to residual immunity against severe outcomes acquired from vaccination and prior infection.

**Key public health message**

**What did you want to address in this study?**

Less than 1 month after first being detected in Italy in November 2021, the Omicron variant became the most prevalent SARS CoV-2 variant in the country. We wished to understand how quickly Omicron spread in Italy and how transmissible the new variant is.

**What have we learnt from this study?**

Between December 2021 and January 2022, the Omicron emergence caused a marked and rapid increase in SARS-CoV-2 infections and an increase in COVID-19 cases while a lower increase in hospitalisations was observed, and intensive care use remained stable.

**What are the implications of your findings for public health?**

Our estimates indicate that the Omicron variant disseminates faster in the population than the Delta variant. Among those who get infected, a higher proportion has no symptoms and fewer require intensive care, possibly because by the time Omicron became dominant, many people were already protected from severe disease by vaccination or earlier infections with other variants.

## Introduction

The severe acute respiratory syndrome coronavirus 2 (SARS-CoV-2) variant of concern (VOC) Omicron (Phylogenetic Assignment of Named Global Outbreak (Pango) lineage designation: B.1.1.529), characterised by a large number of mutations in the spike gene, has shown a marked growth advantage over pre-circulating lineages, causing a major upsurge of cases in multiple countries from November 2021 onwards [1]. As Europe was transitioning rapidly from Delta (Pango lineage: B.1.617.2) to Omicron dominance, data on presence and proportion of VOCs among SARS-CoV-2 infections across distinct geographical contexts and over time supported assessments of the current and potential short-term implications for public health.

Here, we robustly assess the temporal expansion of Omicron variant in Italy by analysing data collected during four rapid genomic surveys conducted in the country between 6 December 2021 and 17 January 2022. Results are combined with data gathered by the Italian Integrated Surveillance System with the aim of estimating the impact of the Omicron spread on the upsurge of SARS-CoV-2 transmission and of hospital admissions.

## Methods

### Genomic surveys and sequencing

Genomic surveys were conducted on 6 and 20 December 2021, and on 3 and 17 January 2022. The initiative involved all 19 Regions and two Autonomous Provinces (APs) of Italy and was coordinated by the National Public Health Institute (Istituto Superiore di Sanità, ISS), in collaboration with the Ministry of Health, the Bruno Kessler Foundation, and 120 laboratories distributed across the country.

On the dates of the surveys, random samples were selected in each Region/AP among infections diagnosed with a real-time reverse-transcription PCR (RT-PCR). The sample size was calculated to enable the detection of circulating SARS-CoV-2 variants with a proportion of at least 5% within each of four macro-areas (North-East, North-West, Centre, and South/Islands) with a 2% precision (Supplementary Material section 1). Samples were subjected to whole genome sequencing (WGS) as the gold standard method for variant detection. Alternatively, results from Sanger or next generation sequencing (NGS) of the whole or partial S-gene were collected. Sequences with insufficient quality for variant assignment were discarded from the analysis.

### Assessments of Omicron proportion over time

To robustly assess the temporal expansion of Omicron, the proportion of this variant was estimated in each Region/AP using two alternative approaches. The first used Markov Chain Monte Carlo (MCMC) under the assumption of independence in proportion across Regions/APs and surveys. The second fitted a generalised linear mixed model (GLMM) assuming a random intercept and a random slope for each Region/AP; the independent variable was the day when the survey took place (Supplementary Material section 2). A binomial distribution for the identified number of Omicron sequences was assumed in both approaches. The procedures were replicated on data aggregated by macro-area.

### Estimation of doubling time and net reproduction number

The approximate number of Omicron cases in each Region/AP over time was obtained by multiplying the estimated local proportion from the surveys by the corresponding number of cases notified to the Italian Integrated Surveillance System. Estimates obtained for the first three survey dates were aggregated at the national level and fitted with an exponential curve having growth rate *r*. The latter was used to estimate the doubling time of Omicron as *T = log(2)/r* and the net reproduction number as *R = 1 + r × GT*, where *GT* represents the average generation time. In the absence of estimates of *GT* for Omicron, we considered values between 4 and 8 days, covering a range of estimates for previous lineages [2-6] (Supplementary Material section 3). The same procedure was applied to data collected during the first two surveys only for sensitivity analysis.

### Analysis of epidemiological surveillance data

The upsurge of SARS-CoV-2 transmission in Italy due to the Omicron spread was additionally evaluated by estimating daily SARS-CoV-2 net *R* values using data collected by the Italian Integrated Surveillance System [7]. Separate estimates were obtained from the time series of symptomatic cases by date of symptom onset (sym; *R_sym_*), and from the time series of patients admitted to hospital (hos, *R_hos_*) and to intensive care units (ICU; *R_ICU_*) by date of admission. Estimates of the net reproduction numbers are based on the assumption that the Omicron generation time is comparable to that of pre-circulating strains [2,8,9]. The same generation time was assumed to compute reproduction numbers from time series of symptomatic cases, hospitalisations, and ICU admissions. Methodological details are described in the Supplementary Material section 4. Additional information on data routinely collected within the Italian Integrated Surveillance System and applied case definitions can be found in an earlier report [7].

## Results

On 6 December 2021, 2,241 samples were collected and 2,127 successfully sequenced. Of these, four harboured Omicron (0.2% of samples), 2,121 Delta (99.7%, Table 1), and two had B.1.640 and Q.4 viruses respectively (0.1%, Table 1). Omicron was only found in Northern Italy (Emilia-Romagna, Lombardy, and Veneto). Estimates of the national proportion of Omicron among circulating variants of SARS-CoV-2 ranged on average from 0.5% to 1.8% (Table 2).

**Table 1:** Results of genomic surveys conducted across all 21 participating Regions/Autonomous Provinces, Italy, December 2021 (n = 4,266 analysed sequences)

MACRO-AREA	REGION^a^	6 December 2021								20 December 2021							
		Number of				CONFIRMED CASES				Number of				CONFIRMED CASES			
		LABs	RT-PCR POSITIVE	SEQUENCED SAMPLES	ANALYSED SAMPLES	Delta		Omicron		LABs	RT-PCR POSITIVE	SEQUENCED SAMPLES	ANALYSED SAMPLES	Delta		Omicron	
						N	%^b^	N	%^b^					N	%^b^	N	%^b^
SOUTH/ ISLANDS	ABR	2	89	55	55	55	NA	0	NA	2	179	63	62	52	83.9	10	16.1
	APU	11	53	53	53	53	NA	0	NA	11	59	57	57	44	NA	13	NA
	BAS	2	7	7	7	7	NA	0	NA	2	131	10	10	5	NA	5	NA
	CAL	4	224	45	39	39	NA	0	NA	4	475	80	71	69	97.2	2	2.8
	CAM	3	150	150	140	140	100.0	0	0.0	3	1,770	134	131	114	87.0	17	13.0
	MOL	1	1	1	1	1	NA	0	NA	1	7	7	7	6	NA	1	NA
	SAR	10	223	30	30	30	NA	0	NA	10	338	31	30	25	NA	5	NA
	SIC	4	209	159	159	159	100.0	0	0.0	5	354	246	246	203	82.5	43	17.5
**TOTAL SOUTH**		**37**	**956**	**500**	**484**	**484**	**100.0**	**0**	**0.0**	**38**	**3,313**	**628**	**614**	**518**	**84.4**	**96**	**15.6**
CENTRE	LAZ	5	515	511	435c	434	99.8	0	0.0	6	362	339	318d	262	82.4	55	17.3
	MAR	5	62	62	62	62	100.0	0	0.0	5	92	68	67	63	94.0	4	6.0
	TUS	3	155	73	71	71	100.0	0	0.0	3	260	84	84	42	50.0	42	50.0
	UMB	4	164	44	44	44	100.0	0	0.0	4	498	68	68	24	35.3	44	64.7
**TOTAL CENTRE**		**17**	**896**	**690**	**612**	**611**	**99.8**	**0**	**0.0**	**18**	**1,212**	**559**	**537**	**391**	**72.8**	**145**	**27.0**
NORTH-EAST	BOL	1	61	58	58	58	NA	0	NA	1	94	45	45	43	NA	2	NA
	TRE	1	15	15	14	14	NA	0	NA	1	17	17	17	13	NA	4	NA
	EMI	3	106	106	106	105	99.1	1	0.9	3	133	133	133	111	83.5	22	16.5
	FRI	6	377	150	146	146	100.0	0	0.0	6	254	60	59	54	NA	5	NA
	VEN	12	234	234	234	232	99.1	2	0.9	12	219	219	219	201	91.8	18	8.2
**TOTAL NORTH-EAST**		**23**	**793**	**563**	**558**	**555**	**99.5**	**3**	**0.5**	**23**	**717**	**474**	**473**	**422**	**89.2**	**51**	**10.8**
NORTH-WEST	AOV	1	3	3	3c	2	NA	0	NA	1	5	5	5	3	NA	2	NA
	LIG	9	247	57	53	53	NA	0	NA	10	610	52	48	42	NA	6	NA
	LOM	16	346	332	324	323	99.7	1	0.3	16	460	350	339	202	59.6	137	40.4
	PIE	11	96	96	93	93	100.0	0	0.0	11	126	126	123	98	79.7	25	20.3
**TOTAL NORTH-WEST**		**37**	**692**	**488**	**473**	**471**	**99.6**	**1**	**0.2**	**38**	**1,201**	**533**	**515**	**345**	**67.0**	**170**	**33.0**
TOTAL ITALY		114	3,337	2,241	2,127^c^	2,121	99.7	4	0.2	117	6,443	2,194	2,139^d^	1,676	78.4	462	21.6

AP: Autonomous Province; Delta: Pango lineage B.1.617.2; labs: laboratories; NA: non applicable (the sample size is less than 60); Omicron: Pango lineage B.1.1.529; Pango: Phylogenetic Assignment of Named Global Outbreak; RT-PCR: real-time reverse-transcription PCR.

^a^ ABR: Abruzzo; AOV: Aosta valley; APU: Apulia; BAS: Basilicata; BOL: AP Bolzano; CAL: Calabria; CAM: Campania; EMI: Emilia-Romagna; FRI: Friuli Venezia Giulia; LAZ: Lazio; LIG: Liguria; LOM: Lombardy; MAR: Marche; MOL: Molise; PIE: Piedmont; SAR: Sardinia; SIC: Sicily; TRE: AP Trento; TUS: Tuscany; UMB: Umbria; VEN: Veneto.

^b^ Percentages represent the fraction of confirmed infections over the total analysed samples.

^c^ Lazio notified one B.1.640 sequence and Aosta valley one Q.4 sequence.

^d^ Lazio notified one B.1.640 sequence.

**Table 2:** National level estimates from surveys data for the Omicron proportion, exponential growth rate, doubling time and net reproduction number, Italy, 6 December 2021–17 January 2022

Parameters		GLMM		MCMC	
		Applied to regional data	Applied to macro-area data	Applied to regional data	Applied to macro-area data
		Mean (95% CrI)	Mean (95% CrI)	Mean (95% CrI)	Mean (95% CrI)
**National ** **proportion** (%)	**6 December 2021**	1.6 (1.3–2.1)	1.8 (1.4–2.2)	1.4 (0.9–2.1)	0.5 (0.2–0.9)
	**20 December 2021**	20.0 (18.4–21.6)	20.6 (18.9–22.2)	21.5 (19.7–23.5)	21.5 (19.8– 23.2)
	**3 January 2022**	78.4 (76.8–79.9)	76.9 (75.4–78.5)	80.2 (78.6–81.9)	80.1 (78.6–81.5)
	**17 January 2022**	97.8 (97–98.5)	97.7 (96.9–98.4)	94.6 (93.6–95.6)	94.6 (93.6–95.5)
***r*** (days ^− 1^)		0.23 (0.17–0.29)	0.23 (0.17–0.28)	0.22 (0.16–0.28)	0.26 (0.18–0.35)
***T*** (days)		3.1 (2.44–4.00)	3.13 (2.46–4.05)	3.27 (2.51–4.29)	2.73 (2.01–3.79)
***R* if *GT* = 4 days**		1.91 (1.69–2.14)	1.90 (1.68–2.13)	1.86 (1.65–2.10)	2.04 (1.73–2.38)
***R* if *GT* = 6 days**		2.37 (2.04–2.71)	2.35 (2.03–2.69)	2.30 (1.97–2.65)	2.56 (2.10–3.07)
***R* if *GT* = 8 days**		2.82 (2.39–3.28)	2.80 (2.37–3.26)	2.73 (2.29–3.21)	3.08 (2.46–3.76)

CrI: credible interval; GLMM: generalised linear mixed model; *GT*: generation time; MCMC: Markov Chain Monte Carlo; Omicron: Phylogenetic Assignment of Named Global Outbreak (Pango) lineage B.1.1.529; *r*: growth rate of Omicron infections; *R*: net reproduction number; *T*: doubling time.

On 20 December 2021, 2,194 samples were collected and 2,139 successfully sequenced; 462 resulted Omicron (21.6%) and 1,676 Delta (78.4%) positive; one sequence was associated with B.1.640 (0.0%, Table 1). Omicron was detected in all Regions/APs, and it was the predominant variant in Umbria (Central Italy). Estimates of the national proportion ranged on average from 20% to 21.5% (Table 2).

By 3 January 2022 (2,632 samples, 2,571 successfully sequenced), Omicron had become largely dominant in the country, being confirmed in 2,058 (80.0%, Table 3) sequences (Delta: 512 sequences, 19.9%; B.1.639: 1 sequence, 0.1%). Estimates of the national proportion of Omicron ranged on average from 76.9% to 80.2% (Table 2).

**Table 3:** Results of genomic surveys conducted across all 21 participating Regions/Autonomous Provinces, Italy, January 2021 (n = 4,948 analysed sequences)

MACRO-AREA	REGION^a^	3 January 2022								17 January 2022							
		Number of				CONFIRMED CASES				Number of				CONFIRMED CASES			
		LABS	RT-PCR POSITIVE	SEQUENCED SAMPLES	ANALYSED SAMPLES	Delta		Omicron		LABS	RT-PCR POSITIVE	SEQUENCED SAMPLES	ANALYSED SAMPLES	Delta		Omicron	
						N	%^b^	N	%^b^					N	%^b^	N	%^b^
SOUTH/ ISLANDS	ABR	2	1,990	117	117	26	22.2	91	77.8	2	1,521	40	40	3	NA	37	NA
	APU	11	76	74	74	6	8.1	68	91.9	11	37	37	37	0	NA	37	NA
	BAS	2	535	11	11	0	NA	11	NA	2	482	9	9	0	NA	9	NA
	CAL	4	1,073	40	35	10	NA	25	NA	3	1,693	45	30	1	NA	29	NA
	CAM	3	9,802	221	213	62	29.1	151	70.9	3	4,331	227	220	20	9.1	200	90.9
	MOL	1	46	46	46	1	NA	45	NA	1	32	11	11	0	NA	11	NA
	SAR	10	1,237	54	54	9	NA	45	NA	10	1,576	52	52	2	NA	50	NA
	SIC	5	1,807	274	274	58	21.2	216	78.8	5	841	207	207	27	13.0	180	87.0
**TOTAL SOUTH**		**38**	**16,566**	**837**	**824**	**172**	**20.9**	**652**	**79.1**	**37**	**10,513**	**628**	**606**	**53**	**8.7**	**553**	**91.3**
CENTRE	LAZ	4	407	345	301	44	14.6	257	85.4	5	529	529	461	17	3.7	444	96.3
	MAR	5	82	50	50	9	18.0	41	82.0	5	76	48	48	1	NA	47	NA
	TUS	3	276	139	139	15	10.8	124	89.2	3	312	156	156	1	0.6	155	99.4
	UMB	5	1,036	91	90	6	6.7	84	93.3	4	541	48	48	0	NA	48	NA
**TOTAL CENTRE**		**17**	**1,801**	**625**	**580**	**74**	**12.8**	**506**	**87.2**	**17**	**1,458**	**781**	**713**	**19**	**2.7**	**694**	**97.3**
NORTH-EAST	BOL	1	371	24	24^c^	10	NA	13	NA	1	281	40	40^d^	3	NA	36	NA
	TRE	1	26	26	25	6	NA	19	NA	1	23	23	18	3	NA	15	NA
	EMI	3	131	131	131	27	20.6	104	79.4	3	197	197	193	2	1.0	191	99.0
	FRI	8	422	96	96	34	35.4	62	64.6	8	201	70	67	2	3.0	65	97.0
	VEN	13	316	316	316	107	33.9	209	66.1	13	179	179	179	8	4.5	171	95.5
**TOTAL NORTH-EAST**		**26**	**1,266**	**593**	**592**	**184**	**31.1**	**407**	**68.8**	**26**	**881**	**509**	**497**	**18**	**3.6**	**478**	**96.2**
NORTH-WEST	AOV	1	3	3	3	2	NA	1	NA	1	5	5	5	0	NA	5	NA
	LIG	8	815	30	30	7	NA	23	NA	12	2,496	45	45	2	NA	43	NA
	LOM	16	443	443	443	50	11.3	393	88.7	16	396	396	392	18	4.6	374	95.4
	PIE	14	101	101	99	23	23.2	76	76.8	15	122	122	119	4	3.4	115	96.6
**TOTAL NORTH-WEST**		**39**	**1,362**	**577**	**575**	**82**	**14.3**	**493**	**85.7**	**44**	**3,019**	**568**	**561**	**24**	**4.3**	**537**	**95.7**
TOTAL ITALY		120	20,995	2,632	2,571^c^	512	19.9	2,058	80.0	124	15,781	2,486	2,377^d^	114	4.8	2,262	95.2

AP: Autonomous Province; Delta: Pango lineage B.1.617.2; labs: laboratories; NA: non applicable (the sample size is less than 60); Omicron: Pango lineage B.1.1.529; Pango: Phylogenetic Assignment of Named Global Outbreak; RT-PCR: real-time reverse-transcription PCR.

^a^ ABR: Abruzzo; AOV: Aosta valley; APU: Apulia; BAS: Basilicata; BOL: AP Bolzano; CAL: Calabria; CAM: Campania; EMI: Emilia-Romagna; FRI: Friuli Venezia Giulia; LAZ: Lazio; LIG: Liguria; LOM: Lombardy; MAR: Marche; MOL: Molise; PIE: Piedmont; SAR: Sardinia; SIC: Sicily; TRE: AP Trento; TUS: Tuscany; UMB: Umbria; VEN: Veneto.

^b^ Percentages represent the fraction of confirmed infections over the total analysed samples. They are reported as NA when the sample size is less than 60.

^c^ AP Bolzano notified one B.1.639 sequence.

^d^ AP Bolzano notified one B.1.639 sequence.

As of 17 January 2022 (2,486 samples, 2,377 successfully sequenced), Omicron has outcompeted all other circulating strains in the country, being confirmed in 2,262 (95.2%, Table 3) sequences (Delta: 114 sequences, 4.8%; B.1.639: one sequence, 0.0%). Estimates of the national proportion ranged on average from 94.6% to 97.8% (Table 2). 

**Figure 1:**
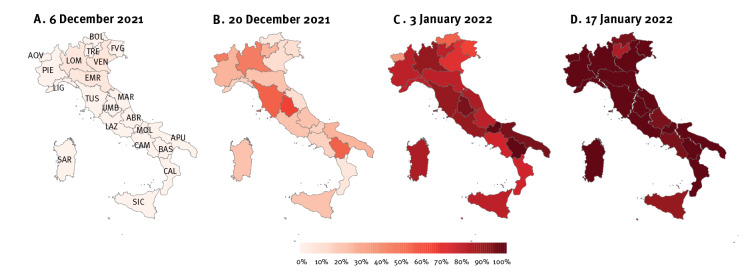
Geographical distribution of SARS-CoV-2 variant Omicron, Italy, 6 December 2021–17 January 2022

ABR: Abruzzo; AOV: Aosta valley; APU: Apulia; BAS: Basilicata; BOL: Autonomous Province Bolzano; CAL: Calabria; CAM: Campania; EMI: Emilia-Romagna; FRI: Friuli Venezia Giulia; LAZ: Lazio; LIG: Liguria; LOM: Lombardy; MAR: Marche; MOL: Molise; PIE: Piedmont; SAR: Sardinia; SIC: Sicily; TRE: Autonomous Province Trento; TUS: Tuscany; UMB: Umbria; VEN: Veneto.

Results for the four surveys are reported in Figure 1, and in Tables 1 and 3. Corresponding proportion estimates are reported in Table 2.

Point estimates of the proportion of Omicron (Phylogenetic Assignment of Named Global Outbreak lineage (Pango) lineage B.1.1.529) by the 19 Regions and two Autonomous Provinces of Italy as obtained from national surveys conducted on 6 December 2021, 20 December 2021, 3 January 2022 and 17 January 2022. Regions with their abbreviated names are shown in the leftmost panel.

By using proportion estimates retrieved for the first three dates, we estimated an average daily exponential growth rate *r* for new Omicron infections of 0.22–0.26 days ^− 1^, corresponding to an average doubling time of 2.7–3.3 days and reproduction numbers in the range 1.86–2.04 and 2.73–3.08, when a generation time of 4 and 8 days was assumed respectively (Table 2). Similar estimates were obtained when the exponential growth was fitted on the first two surveys only (Supplementary Table S2).

**Figure 2:**
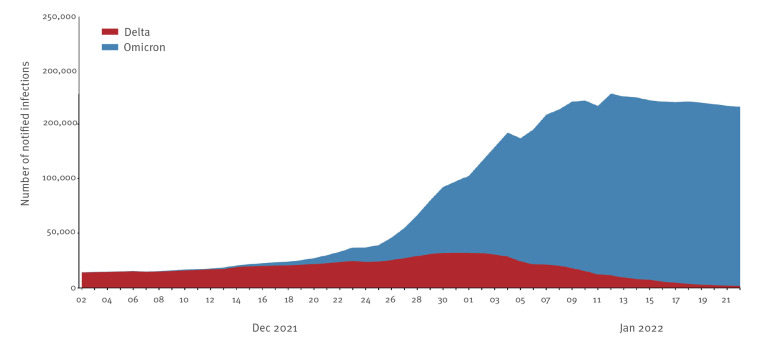
Estimates of notified infections attributable to Omicron and Delta variants over time, Italy, 02 December 2021–22 January 2022

We note that, despite the decrease in Delta proportion, the average total number of estimated Delta cases also increased in the observation period from 9,336–9,458 on 6 December 2021, to 13,455–15,701 on 3 January 2022 (Supplementary Table S1 and Figure 2). In the subsequent period, Delta cases decreased to 1,844–4,521 on 17 January.

Delta: Phylogenetic Assignment of Named Global Outbreak (Pango) lineage B.1.617.2; Omicron: Pango lineage B.1.1.529.

Number of daily Omicron (moving average over 7 days in blue) and Delta (moving average over 7 days in red) infections as obtained by combining the time series of infections ascertained across different Regions and Autonomous Provinces of Italy with average local proportion as estimated by applying a generalised linear mixed model to observed data.

**Figure 3:**
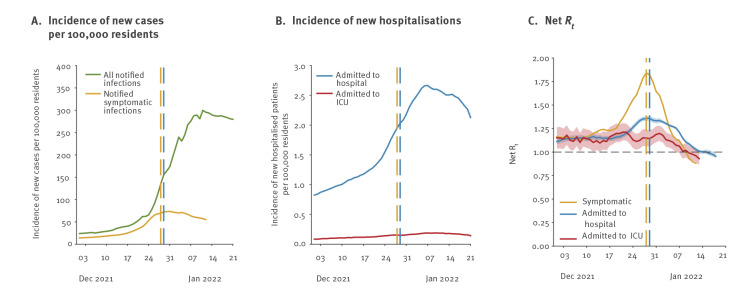
The impact of Omicron spread on SARS-CoV-2 circulation, Italy, December 2021–January 2022

ICU: intensive care unit; Omicron: Phylogenetic Assignment of Named Global Outbreak (Pango) lineage B.1.1.529; SARS-CoV-2: severe acute respiratory coronavirus 2.

The impact of Omicron expansion on the overall SARS-CoV-2 circulation was quantified by estimating reproduction numbers from the time series of cases recorded by the Italian Integrated Surveillance System. We found that *R_sym_* increased from 1.15 (95% credible interval (CrI): 1.15–1.15) on 6 December 2021 to a peak of 1.83 (95%CrI: 1.83–1.84) on 28 December (Figure 3). *R_hos_* increased from 1.14 (95%CrI: 1.12–1.18) on 6 December 2021, to a peak of 1.36 (95%CrI: 1.33–1.38) on 29 December 2021. In contrast, *R_ICU_* did not show a clear temporal trend throughout December 2021, with mean estimates ranging between 0.93 and 1.21.

A. Time series of total number of notified and symptomatic cases by date of notification and symptom onset (moving average over 7 days), respectively. The number of symptomatic cases by date of symptom onset is shown only for data consolidated on 21 January 2022.

B. Time series of total number of cases admitted to hospital and to ICU, by date of admission (moving average over 7 days).

C. Net reproduction numbers *R_t_* for symptomatic cases, cases admitted to hospital and to ICU, respectively in yellow, blue and red. The solid line represents the mean of the estimated posterior distribution, and the shaded areas delimit 95% credible intervals.

The yellow and blue dashed vertical lines represent the days at which the net reproduction numbers for symptomatic and hospitalised cases respectively reach their peak values, as shown in panel C.

However, a progressive decline of all reproduction numbers was observed in the first weeks of 2022, yielding their mean estimates under the epidemic threshold of 1 between 10 and 16 January.

## Discussion

Omicron was first identified in Italy in the second half of November 2021 [10]. Four genomic surveys were successively conducted to evaluate the progressive spread of the variant in the country. The presented results show that Omicron variant (BA.1 subvariant at that time) became dominant across the Italian territory in less than 1 month, significantly increasing SARS-CoV-2 transmission.

Our estimates for the doubling time (2.7–3.3 days) are in agreement with those obtained in other countries [11,12]. The reproduction number associated to the Omicron variant under the transmissibility conditions existing in Italy in December may be in the range of 1.8–3.1. This estimate is compatible with values of *R_sym_* around 1.8 observed on 28 December 2021, when the replacement of Delta by Omicron was not complete and before various control measures (introduced on 24 and 30 December [13,14]) and behaviour change had a chance to significantly affect transmission. Notably, changes in contact patterns due to the Christmas holidays may have had an effect that is hard to quantify.

The selective advantage of Omicron over Delta may be explained by increased transmissibility, partial immune escape, or a combination of both [15]. Early evidence from statistical analysis of reinfections and breakthrough infections suggested a marked capability of escape from natural and vaccine-acquired immunity [11,16-19]. However, further efforts are needed to quantify the relative transmissibility of Omicron compared with Delta, and its inherent capacity to cause severe disease, which is key to evaluate potential changes in COVID-19 burden potentially led by this and the progressive waning of immunity.

The lower increase of reproduction numbers associated to hospital admissions and the stable value of those from ICU admissions may suggest, under the epidemiological conditions observed in late 2021, a reduced severity of Omicron compared with Delta. However, while only partially protected against infection, individuals who received at least two vaccine doses were substantially protected against severe outcomes [20,21]. It is therefore likely that the lower disease severity observed in populations during the Omicron wave can be partially ascribed to the protection conferred by vaccination and/or prior infection [22,23], explaining the large decoupling between identified cases and hospitalised individuals.

Marked changes in the ascertainment rate of SARS-CoV-2 infections may have also occurred during the study period due to the brisk increase of identified cases and the consequent saturation of the testing system, the reduced likelihood of test-seeking given the infection, and the possible increase of voluntary testing in preparation for gatherings during Christmas and New Year’s holidays [24]. On the other hand, the widespread transmission of Omicron observed throughout January 2022 could have inflated the number of patients admitted to hospital and ICU with a positive test for SARS-CoV-2 infection but with a different primary reason for hospitalisation. Changes in the ascertainment rate of SARS-CoV-2 infection and COVID-19 patients should have a limited impact on the estimated proportion of circulating lineages, and on the growth rate of Omicron infections. However, they may have affected estimates of the reproduction number from different data sources during the replacement of pre-circulating linages by Omicron. All these factors prevented us to quantify the relative risk of hospitalisation and of ICU admission for Omicron compared with Delta.

Of note, we estimate that the total number of Delta cases increased over December 2021, suggesting that a considerable fraction of hospital patients recorded in early January could be attributed to Delta. As such, continued monitoring of Delta proportion among severe cases was recommended.

The four daily surveys were based on relatively small sample sizes and, despite the randomisation of sampling for genomic sequencing, biases due to the presence of large clusters of cases in a region cannot be excluded. Moreover, estimates of the net reproduction numbers are based on the assumption that the Omicron generation time is comparable to that of pre-circulating strains, as suggested by recent estimates for the Italian context [8,9].

Efforts combining genomic and epidemiological surveillance for tracking circulating variants and analysing epidemiological trends at the population level are of the utmost importance to support public health responses and improve system preparedness for future epidemic threats.

## Supplementary Data

228000 Supplementary Material
