# Supplementary material for: Tracking the progressive spread of the SARS-CoV-2 Omicron variant in Italy, December 2021 to January 2022
Source: Euro Surveill. 2022 Nov 10;27(45):2200125. doi: 10.2807/1560-7917.ES.2022.27.45.2200125 (PMC9650705; doi:10.2807/1560-7917.ES.2022.27.45.2200125)
Supplement: Supplementary Material [file 2200125_SupplementaryMaterial.pdf]

Supplement

Tracking the progressive spread of the SARS-CoV-2 Omicron variant in Italy, December 2021 to January 2022.

This supplementary material is hosted by Eurosurveillance as supporting information alongside the article "Tracking the progressive spread of the SARS-CoV-2 Omicron variant in Italy, December 2021 – January 2022.", on behalf of the authors, who remain responsible for the accuracy and appropriateness of the content. The same standards for ethics, copyright, attributions and permissions as for the article apply. Supplements are not edited by Eurosurveillance and the journal is not responsible for the maintenance of any links or email addresses provided therein.

Table of Contents

1. Sample size estimation for genomic surveillance **2**

2. Estimation of Omicron proportion and notified cases from surveys data **2**

a. Monte Carlo Markov Chain approach 2

b. Regression modelling approach 3

3. Estimation of Omicron exponential growth rate, doubling time and net reproduction number (R) from surveys data **4**

4. Estimation of the SARS-CoV-2 net reproduction numbers (Rt) from surveillance of cases **4**

5. Software **5**

References **6**

1. Sample size estimation for genomic surveillance

Genomic surveillance in Italy is coordinated by the Italian National Institute of Health, in collaboration with the Ministry of Health and the laboratories of Regions/Autonomous Provinces (AP). Within the context of rapid detection and surveillance of emerging variants of concern (VOC), ad-hoc surveys are carried out in Italy. From the emergence of the VOC Omicron, four genomic surveys were conducted on December 6, 2021, December 20, 2021, January 3 and January 17, 2022, to estimate its prevalence. As of December 6, Delta was the predominant SARS-CoV-2 lineage in Italy [1].

The surveys involved all the 19 Regions and 2 Autonomous Provinces (AP) of Italy. Random samples of SARS-CoV-2 positive cases were analyzed by 120 laboratories distributed across the national territory. Samples were selected from both passive surveillance and contact tracing and included symptomatic, pre-symptomatic and asymptomatic cases. The collected samples were sequenced according to the local laboratory policy by either of the following techniques: i) sequencing the entire S-gene by Sanger technology, ii) sequencing part of the S-gene with the identification of all mutations/deletions associated with the three variants, or iii) sequencing the whole genome by Next Generation Sequencing.

Samples were distributed across 4 macro-areas, defined according to the Eurostat NUTS1 classification: North-East, North-West, Center, and South/Islands. The sample size was calculated to have the statistical power to detect a prevalence of 5%, with precision 2%, within each macro-area, based on the following equation

$$n \geq \frac{\mathrm{N} z_{\alpha/2}^{2}\, \mathrm{p}(1-\mathrm{p})}{\varepsilon^{2}(\mathrm{N}-1)+z_{\alpha/2}^{2}\mathrm{p}(1-\mathrm{p})}$$

where N is the total number of cases notified on the day preceding each of the three surveys [2], p is the target prevalence (5%), ε is the desired precision (2%), α is the significance level set at 5%, and $z_{\alpha/2}^{2}$ the corresponding z-score (1.96).

2. Estimation of Omicron proportion and notified cases from surveys data

To estimate the Omicron proportion and the number of notified cases, we employed two alternative approaches that are described below.

a. Monte Carlo Markov Chain approach

We fitted the Omicron proportion in the three surveys by means of a Markov Chain Monte Carlo (MCMC) approach applied to the binomial likelihood of observing the identified number of omicron infections among the sequenced genomes under the assumption that each geographical unit as well as each survey is independent. The MCMC approach was applied considering two different subdivisions of the Italian territory. In the first one, we consider regions as the observational unit; in the second one, the 4 macro-areas defined in the previous section. We fitted 10000 iterations of MCMC separately for each unit (either region or macro-area) and for each survey. We then approximated the local number of Omicron cases by multiplying the obtained proportion with the corresponding number of cases notified at the date of sample collection. Secondary outcomes are *i)* the distributions of the number of notified Omicron cases in each unit and in the whole Italy (obtained by addition of local cases) and *ii)* the distributions of Omicron proportion in the whole Italy, obtained by dividing the estimated number of Omicron cases by the total number of cases notified on 6 December 2021, 20 December 2021, 3 January 2022 and 17 January 2022. The same approach was used to approximate the number of notified Delta cases over the three survey days. Results are reported in the main text and in Tables S1.

b. Regression modelling approach

We fitted a generalized linear mixed model to estimate Omicron proportion when the three surveys were conducted under the assumption that observed variation among geographical unit (both in initial proportion and daily increase) are normally distributed around an average national value and that exist a correlation between surveys results within each region. We applied a Bayesian approach for the estimation of model parameters. The model is applied considering two different subdivisions of the Italian territory. In the first one, we consider regions as the observational unit; in the second one, we aggregated regional data in the 4 macro-areas defined in the previous section. In both cases, we assumed a binomial distribution for the dependent variable, represented by the total number of omicron sequences out of the total number of sequences analyzed in each unit (either region or macro-area). We considered as independent variable the date when surveys were carried out. Dates were expressed as the number of days elapsed from the first survey: 6 December 2021 corresponds to day 0; 20 December 2021 to day 14; 3 January 2022 to day 28: 17 January 2022 to day 42. For computational convenience, we rescaled the day variable by dividing it by its maximum value. We considered a random intercept and a random slope for each unit (either region or macro-area) to allow for heterogeneities among units both in the detected initial proportion and in the growth rate of Omicron proportion. The adopted model equation was:

$$
\begin{gathered}
y_{i,s} \sim Bin(p_{i,s}, S_{i,s}) \\
logit(p_{i,s}) = \alpha + a_i + (\beta + b_i)\, day_s \\
\alpha \sim Norm(-5, 0.1) \\
\beta \sim Norm(5, 1000) \\
a_i \sim half - Cauchy(25) \\
b_i \sim half - Cauchy(25)
\end{gathered}
$$

where the subscript i and s identify the geographical unit and the survey considered, respectively; α is the intercept, representing the average proportion among units, and is assumed to follow an informative Normal prior of parameters mean -5 and variance 0.1; β is the slope of the regression model, representing the average rate of change in proportion by day among units, and it is assumed to follow a non-informative Normal prior distribution of parameters mean 5 and variance 1000; a_i and b_i are the random effect coefficients for the intercept and the slope, respectively, and are assumed to follow a half-Cauchy prior of parameter 25; day_s represents the number of days from the first survey to survey s (which is the same for all units i). $S_{i,s}$ represents the total number of sequences analyzed in unit i during survey s; $y_{i,s}$ is the number of Omicron sequences confirmed in unit i during survey s and $p_{i,s}$ is the estimated Omicron proportion in unit i during survey s. Three chains were used in the MCMC process with a burn-in of 100000 iteration and a thinning rate of 50 resulting in 3000 iterations used to estimate each posterior distribution.

The posterior distributions of the number of notified Omicron cases in each observational unit (either region or macro-area depending on the model) and in the entire Italy on 6 December 2021, 20 December 2021, 3 January 2022 and 17 January 2022 were obtained by multiplying realizations from the estimated posterior distribution of Omicron proportion with the corresponding number of cases notified at the date of sample collection. The posterior distributions of the number of notified Omicron cases in Italy was then obtained by adding the estimates computed for each observational unit. The same approach was used to approximate the number of notified Delta cases over the three survey days. Results are reported in the main text and in Table S1.

3. **Estimation of Omicron exponential growth rate, doubling time and net reproduction number (*R*) from surveys data**

To estimate the growth rate, the doubling time and R associated with Omicron infections and its potential impact on SARS-CoV-2 transmissibility, we applied a linear regression model to a sample (of size 1000) pooled from the posterior distribution of (log-transformed) Omicron cases at national level notified at the date of sample collection. Given the implicit assumption of exponential growth, the model was applied to cases associated with the first three surveys only and, for sensitivity analysis, to those associated with the first two surveys. The posterior distribution of Omicron cases in the first three surveys (two in the sensitivity analysis) was estimated through the approaches described in the previous paragraphs. For each vector of log-transformed cases we applied the following linear regression model:

$$logC_s \sim Norm(\mu_s, \sigma_s^2)$$
$$\mu_s = \eta + \psi \, day_s$$
$$\eta \sim Norm(logC_1, 1)$$
$$\psi \sim Norm((logC_l - logC_1)/day_3, 0.1)$$
$$\sigma_s \sim Gamma(1.5,2)$$

where $logC_s$ is the log-transformed number of notified Omicron cases in Italy on the day of survey *s*, which is assumed to follow a normal distribution of mean μ_s and standard deviation σ_s. We assumed a gamma distribution of shape 1.5 and rate 2 for σ_s. η and ψ are the intercept and the slope in the regression equation, respectively. The slope ψ should be interpreted as the growth rate *r* of the epidemic. We assumed for η an informative normal prior distribution of mean equal to the log-transformed number of notified cases on the day of the first survey and standard deviation equal to 1. We assumed for ψ an informative normal prior distribution of mean equal to the slope of the line intersecting the log-transformed number of notified cases during the day of the first and the third (the second in the sensitivity analysis) survey and standard deviation equal to 0.1. Three chains were used in the MCMC process with a burn-in of 150000 iteration and a thinning rate of 10 resulting in 3000 iterations used to estimate each posterior distribution.
Finally, we computed the posterior distribution of *r* by sampling 1000 values from each one of the posterior distributions of ψ obtained from the pooled 1000 vectors of cases.

The estimated distribution of the exponential growth rate *r* was used to obtain the distribution of the doubling time *T* and of the net reproduction number (*R*) expected after the Omicron expansion in Italy through the following formulas

$$T = \log(2)/r$$
$$R = 1 + r * GT$$

Where GT represents the Omicron generation time (i.e., the average time elapsing between the infection of primary and secondary cases). In the absence of robust estimates for the generation time of Omicron, we considered values of 4, 6 and 8 days.

4. **Estimation of the SARS-CoV-2 net reproduction numbers (*Rt*) from surveillance of cases**

To evaluate the progressive impact of the Omicron expansion on the overall SARS-CoV-2 circulation, we estimated the time varying net reproduction number *Rt* associated to cases reported to the National Integrated Surveillance System. The approach adopted to estimate *Rt* is the same used during the official weekly monitoring of SARS-CoV-2 transmission in Italy. Methodological details can be found in [3].

The net reproduction number quantifies the transmission potential at a given time *t* and is used to monitoring exogenous and endogenous changes in the infection transmission, including those related to performed control measures, changes in the susceptibility and in human behavioral responses to the infection risks.

Estimates of the Omicron exponential growth rate, doubling time and net reproduction number as obtained in the sensitivity analysis are reported in Table S2.

5. Software

All the analysis were performed using the statistical software *R* (version 4.1.0) [4] and *JAGS* (version 4.3.0) [5], and the related packages *R2Jags* [6], *here* [7] and *tidyverse* [8].

Table S1. National level estimates from surveys data for the Omicron and Delta number of cases

		**GLMM**		**MCMC**	
		**Applied to regional data**	**Applied to macro area data**	**Applied to regional data**	**Applied to macro area data**
		**mean (95%CrI)**	**mean (95%CrI)**	**mean (95%CrI)**	**mean (95%CrI)**
**Omicron cases**	**December 6th, 2021**	156 (120 - 198)	167 (129 - 206)	135 (83 - 198)	45 (20 - 82)
	**December 20th, 2021**	3240 (2981 - 3508)	3333 (3069 - 3597)	3478 (3190 - 3784)	3484 (3204 - 3776)
	**January 3rd, 2022**	53320 (52292 - 54349)	52351 (51317 - 53411)	54597 (53457 - 55734)	54513 (53459 - 55549)
	**January 17th, 2022**	81559 (80886 - 82193)	81484 (80845 - 82109)	78927 (78042 - 79707)	78882 (78024 - 79684)
**Delta cases**	**December 6th, 2021**	9347 (9305 - 9383)	9336 (9297 - 9374)	9368 (9302 - 9421)	9458 (9421 - 9483)
	**December 20th, 2021**	12973 (12705 – 13232)	12880 (12616 – 13144)	12735 (12429 – 13023)	12729 (12437 – 13009)
	**January 3rd, 2022**	14732 (13703 - 15760)	15701 (14641 - 16735)	13455 (12318 - 14595)	13539 (12503 - 14593)
	**January 17th, 2022**	1844 (1210 - 2517)	1919 (1294 - 2558)	4476 (3696 - 5361)	4521 (3719 - 5379)

Table S2. Sensitivity analysis considering only the first two surveys when estimating he Omicron exponential growth rate, doubling time, and reproduction number.

	**GLMM**		**MCMC**	
	**Applied to regional data**	**Applied to macro area data**	**Applied to regional data**	**Applied to macro area data**
	**mean (95%CrI)**	**mean (95%CrI)**	**mean (95%CrI)**	**mean (95%CrI)**
**r (days^{-1})**	0.321 (0.182 - 0.461)	0.349 (0.204 - 0.498)	0.234 (0.096 - 0.371)	0.315 (0.172 - 0.457)
**T (days)**	2.26 (1.5 - 3.78)	2.08 (1.39 - 3.38)	2.71 (1.83 - 6.72)	2.36 (1.51 - 3.98)

**R if GT=4 days**	2.29 (1.73 - 2.85)	2.4 (1.82 - 2.99)	1.93 (1.38 - 2.48)	2.26 (1.69 - 2.83)
**R if GT=6 days**	2.93 (2.09 - 3.77)	3.1 (2.22 - 3.99)	2.4 (1.58 - 3.23)	2.89 (2.04 - 3.74)
**R if GT=8 days**	3.57 (2.46 - 4.69)	3.8 (2.63 - 4.98)	2.87 (1.77 - 3.97)	3.52 (2.38 - 4.66)

References

1. Ministero della Salute. Circolare n. 055819 del 03/12/2021 "Stima della prevalenza delle varianti VOC (Variants of Concern) in Italia: beta, gamma, delta, omicron e altre varianti di SARS-CoV-2". Available at https://www.trovanorme.salute.gov.it/norme/renderNormsanPdf?anno=2021&codLeg=84276&parte=1%20&serie=null
2. Dipartimento di Protezione Civile. COVID-19 Italia - Monitoraggio situazione. Data repository at https://github.com/pcm-dpc/COVID-19
3. Riccardo F, Ajelli M, Andrianou XD, Bella A, Del Manso M, Fabiani M, Bellino S, Boros S, Urdiales AM, Marziano V, Rota MC, Filia A, D'Ancona F, Siddu A, Punzo O, Trentini F, Guzzetta G, Poletti P, Stefanelli P, Castrucci MR, Ciervo A, Di Benedetto C, Tallon M, Piccioli A, Brusaferro S, Rezza G, Merler S, Pezzotti P; COVID-19 working group. Epidemiological characteristics of COVID-19 cases and estimates of the reproductive numbers 1 month into the epidemic, Italy, 28 January to 31 March 2020. Euro Surveill. 2020 Dec;25(49):2000790. doi: 10.2807/1560-7917.ES.2020.25.49.2000790.
4. R Core Team (2021). R: A language and environment for statistical computing. R Foundation for Statistical Computing, Vienna, Austria. URL: https://www.Rproject.org/.
5. Plummer, M. (2003). JAGS: A program for analysis of Bayesian graphical models using Gibbs sampling. In Proceedings of the 3rd international workshop on distributed statistical computing (Vol. 124, No. 125.10, pp. 1-10). URL: https://mcmc-jags.sourceforge.io.
6. Yu-Sung Su and Masanao Yajima, (2021). R2jags: Using R to Run 'JAGS'. R package version 0.7-1.
7. Kirill Müller (2020). here: A Simpler Way to Find Your Files. R package version 1.0.1. URL: https://CRAN.R-project.org/package=here.
8. Wickham et al., (2019). Welcome to the tidyverse. Journal of Open Source Software, 4(43), 1686, URL: https://doi.org/10.21105/joss.01686.
